# Cryo-EM structure of human glucose transporter GLUT4

**DOI:** 10.1038/s41467-022-30235-5

**Published:** 2022-05-13

**Authors:** Yafei Yuan, Fang Kong, Hanwen Xu, Angqi Zhu, Nieng Yan, Chuangye Yan

**Affiliations:** 1 State Key Laboratory of Membrane Biology, Beijing Frontier Research Center for Biological Structure, Beijing Advanced Innovation Center for Structural Biology, Tsinghua-Peking Joint Center for Life Sciences, School of Life Sciences, Tsinghua University, Beijing, 100084 China; 2 Present Address: Department of Molecular Biology, Princeton University, Princeton, NJ 08544 USA

**Keywords:** Membrane structure and assembly, Biophysical chemistry

## Abstract

GLUT4 is the primary glucose transporter in adipose and skeletal muscle tissues. Its cellular trafficking is regulated by insulin signaling. Failed or reduced plasma membrane localization of GLUT4 is associated with diabetes. Here, we report the cryo-EM structures of human GLUT4 bound to a small molecule inhibitor cytochalasin B (CCB) at resolutions of 3.3 Å in both detergent micelles and lipid nanodiscs. CCB-bound GLUT4 exhibits an inward-open conformation. Despite the nearly identical conformation of the transmembrane domain to GLUT1, the cryo-EM structure reveals an extracellular glycosylation site and an intracellular helix that is invisible in the crystal structure of GLUT1. The structural study presented here lays the foundation for further mechanistic investigation of the modulation of GLUT4 trafficking. Our methods for cryo-EM analysis of GLUT4 will also facilitate structural determination of many other small size solute carriers.

## Abstract (web summary)

Small solute carriers remain difficult to study by single particle cryo-EM. Here, the authors report the cryo-EM structure of human insulin-responsive glucose transporter GLUT4 (55 kDa) without rigid soluble domains or binders.

## Introduction

Glucose, being the primary fuel, a versatile bio-precursor, and a signaling molecule, is tightly controlled for metabolic homeostasis via various mechanisms, such as hormonal regulation by insulin and glucagon^1^. Insulin lowers blood sugar level by triggering cellular uptake of glucose^2^. The major facilitator superfamily (MFS) glucose transporter GLUT4 mediates the rate-limiting glucose cellular uptake in adipocytes and muscle cells, and thus plays a vital role in insulin-responsive glucose metabolism^3–6^.

At basal state, GLUT4 is primarily distributed on the membranes of the *trans*-Golgi network (TGN), endosomes and 50–70 nm tubulo-vesicular structures known as the GLUT4 storage vesicles (GSVs)^7,8^. Upon insulin stimulation, GLUT4 is quickly transported from these intracellular structures to the plasma membrane, resulting in rapid consumption of glucose from blood^9^. Impairment of cellular glucose uptake due to compromised insulin availability or sensing underlies diabetes mellitus^10^. Therefore, elucidating the structure and working mechanism of GLUT4 will both facilitate our understanding of the fundamental energy metabolism, and shed light on the development of potential intervention strategies for the deleterious disease^11^.

GLUT4, encoded by *SLC2A4*, is one of the 14 members of the SLC2A family. Among the SLC2A members, GLUT4 is most closely related to GLUT1, with a sequence identity and similarity of 65% and 79%, respectively. We solved crystal structures of human GLUT1 and GLUT3 in the inward- and outward-facing conformations a few years ago^12,13^. However, the unique sequences that mediate GLUT4’s membrane trafficking capability, including an FQQI motif on the amino (N) terminus and the LL and TELEY motifs on the carboxyl (C) terminus^14–18^, are not conserved in the SLC2A family, entailing the need to solve the high-resolution structure of GLUT4 (Supplementary Fig. 1).

Despite extensive efforts, we simply could not obtain diffracting crystals for GLUT4. Single particle cryo-electron microscopy (cryo-EM) is more powerful in grappling with low-yield samples with conformational heterogeneity. However, the size of GLUT4, with 509 residues in length, has represented a major technical obstacle for cryo-EM analysis.

**Fig. 1:**
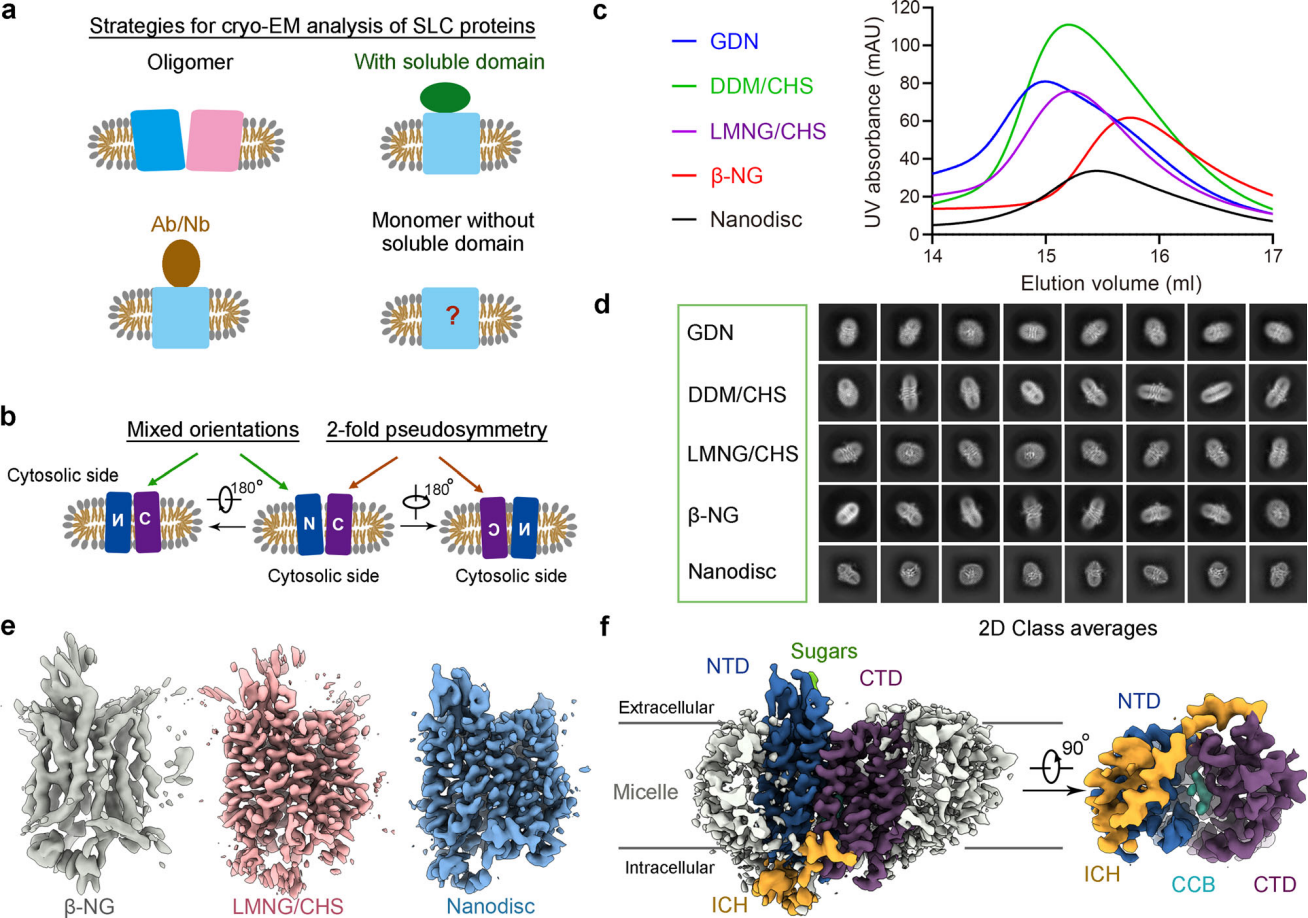
Structural determination of human GLUT4. **a** General strategies for structural resolution of solute carriers (SLCs) using single particle cryo-EM. While homo-oligomeric SLCs are convenient targets for cryo-EM analysis, relatively rigid soluble domains, either intrinsic to the protein, or binders like antibodies (Ab) or nanobodies (Nb), are usually required to increase the size of the protein for cryo-EM imaging. There has been no report on the cryo-EM structure of monomeric SLCs with transmembrane domain only. **b** The two-fold pseudo symmetry of monomeric major facilitator superfamily (MFS) transporters poses another tier of technical challenge for solving their cryo-EM structures. A canonical MFS fold contains 12 transmembrane helices (TMs) organized to an amino terminal domain (NTD) and a C-terminal domain (CTD), which exhibit a two-fold pseudo symmetry around an axis that is perpendicular to the membrane plane. The lack of distinctive soluble domain and the pseudo symmetry imposes serious challenges for discriminating the orientations of the proteins embedded in the disc-like micelles or nanodiscs. **c** Detergent screening for optimal conditions for cryo-EM analysis of human GLUT4. To reduce the impact of the detergent micelles or nanodiscs, we screened for detergents that could yield smaller micelles. Shown here are overlaid SEC profiles of GLUT4 purified in the presence of 0.02% (w/v) GDN, 0.02% (w/v) DDM plus 0.002% (w/v) CHS, 0.01% (w/v) LMNG plus 0.001% (w/v) CHS, 0.1% (w/v) β-NG, and nanodiscs. Source data are provided. **d** Representative 2D class averages of GLUT4 in indicated detergents and nanodiscs. TM features are evident for GLUT4 in LMNG/CHS, β-NG, and nanodiscs. **e** Cryo-EM structural analysis of GLUT4. GLUT4 purified in β-NG gave rise to a decent initial reference and the 3D EM reconstruction was refined to 4.1 Å resolution. 3D EM reconstructions of GLUT4 in LMNG/CHS and nanodisc both reached higher resolutions to 3.3 Å. **f** Overall cryo-EM map of GLUT4 in a nanodisc. The density is domain colored, blue for the NTD, purple for the CTD, yellow for the intracellular helical (ICH) domain, and gray for the micelle. All density and structure figures were prepared in ChimeraX^52^.

In fact, small membrane proteins lacking relatively rigid bulky domains outside the membrane region represent one of the most challenging targets for cryo-EM, as the thick micelles or nanodiscs impede image processing and classification (Fig. 1a). They lack distinct features to facilitate the discrimination of extracellular/luminal and intracellular sides. Proteins with pseudo-symmetries are even more challenging, as the repeats within the membrane cannot be distinguished at low resolution during data processing (Fig. 1b). Recently reported cryo-EM structures of small membrane proteins either have higher symmetry, or consist of a relatively rigid soluble domain^19–23^. To over come these technical barrier, binders like antibodies or nanobodies have to be selected to facilitate cryo-EM analysis^24,25^ (Fig. 1a and Supplementary Table 1).

One of our research interests has been to develop and optimize methods for solving high-resolution structures of small membrane proteins using advanced technology of cryo-EM. We started with proteins containing relatively flexible soluble domains, such as the monocarboxylate transporter MCT1^26^ and the complex of Scap and Insig-2^22,23^. Encouraged by these successful attempts, we took a direct assault on human GLUT4. Here, we report the near atomic resolution cryo-EM structures of GLUT4 bound to a small molecule inhibitor Cytochalasin B (CCB) in detergent micelles and in lipid nanodiscs.

## Results

### Activity characterization and cryo-EM analysis of GLUT4

The full-length wildtype human GLUT4 was fused with an N-terminal Flag tag and transiently expressed in HEK293F cells. After sequential purification through anti-FLAG affinity resin and size-exclusion chromatography (SEC) (Supplementary Fig. 2a), the transport activity of the peak fraction was examined in a proteoliposome-based counterflow assay^13,27^. GLUT4 has a nominal *K*_m_ of 5.4 mM and V_max_ of 3.7 µmol/mg/min for D-glucose transport (Supplementary Fig. 2b). CCB inhibits the glucose transport activity of GLUT4 with an IC_50_ of 3.7 µM (Supplementary Fig. 2c).

As analyzed above, GLUT4, which has a two-fold pseudo symmetry in the membrane and lacks a bulky soluble domain, presented many challenges for cryo-EM analysis. Our previous work with MCT1^26^ and the Scap/Insig-2 complex^22,23^ suggests that the size of the micelles could have a direct impact on 2D and 3D classifications. We therefore set out to screen for detergents that gave rise to the best protein signal (Fig. 1).

We applied GLUT4 to a Superdex 200 column that was pre-equilibrated in the presence of the following detergents, 0.02% (w/v) glyco-diosgenin (GDN), 0.02% (w/v) *n*-dodecyl-β-D-maltoside (DDM) plus 0.002% (w/v) cholesteryl hemisuccinate tris salt (CHS), 0.01% (w/v) lauryl maltose neopentyl glycol (LMNG) plus 0.001% (w/v) CHS, and 0.1% (w/v) *n*-nonyl-β-D-glucopyranoside (β-NG). Indeed, different detergents gave rise to distinct elution peak volumes, earliest at 15 ml with GDN and latest at 15.7 ml with β-NG (Fig. 1c). We also successfully reconstituted the purified GLUT4 into nanodiscs of POPC plus cholesterol surrounded by the membrane scaffold protein MSP1D1. The elution volume of nanodisc-embedded GLUT4 is slightly earlier than β-NG (Fig. 1c).

Next, we examined the proteins purified in different conditions using cryo-EM. To stabilize the structure of GLUT4, 1 mM CCB was added to the purified proteins. Details for cryo-sample preparation and cryo-EM data acquisition are presented in detail in Methods. From the 2D class averages, it is immediately clear that GDN or DDM/CHS results in a weaker protein signal with relatively large and thick micelle. The other three conditions, β-NG, LMNG/CHS, and nanodisc, all present smaller size, and features characteristic of the transmembrane segments (TMs) are discernible (Fig. 1d). We, therefore, proceeded with these three conditions for 3D reconstruction.

For structural determination of small membrane proteins, a good reference map is critical for further classification and refinement. Using Ab-initio construction in cryoSPARC^28^, we were able to obtain a decent initial model for the dataset of GLUT4 in β-NG (Supplementary Fig. 3), but not for that in LMNG/CHS or nanodisc. Details can be found in Methods. Following our previously developed “guided multi-reference 3D classification” and “seed”-facilitated 3D classification strategy^22,23,26,29^, the resolutions of GLUT4 embedded in the LMNG/CHS micelles and nanodiscs both reached 3.3 Å after several rounds of 3D classification and refinement by cryoSPARC (Fig. 1e, Supplementary Figs. 4, 5 and Supplementary Table 2). The excellent EM map allowed for assignment of 464 side chains in the 12 TMs and the intracellular helical (ICH) domain (Fig. 1f, Supplementary Fig. 6, and Supplementary Table 2).

### Inward-open structure of GLUT4 bound to CCB

**Fig. 2:**
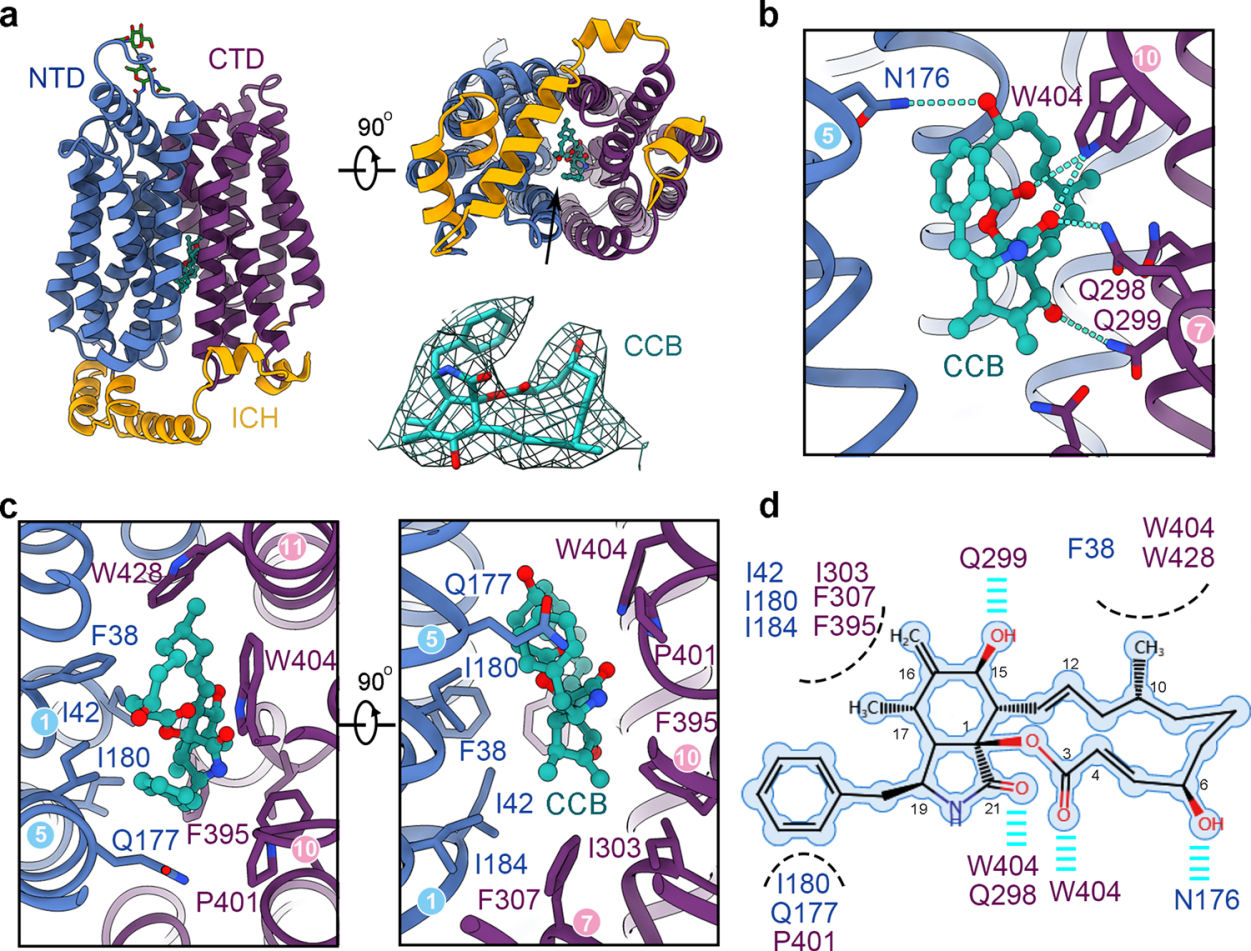
Inward-open structure of GLUT4 bound to the endofacial inhibitor CCB. **a** Cryo-EM structure of GLUT4 in an inward-open state. Two perpendicular views of domain colored GLUT4 are shown. CCB, shown as green ball-and-sticks, binds to the central substrate binding site that is open to the intracellular side. The glycan on the extracellular side is shown as green sticks. The EM density for the bound CCB, shown as green mesh, is contoured at 4 σ. **b** Polar interactions between CCB and GLUT4. Key residues for inhibitor coordination are shown as sticks. The cyan dashed lines indicate hydrogen bonds. **c** Hydrophobic residues surrounding CCB. Two perpendicular views are shown. **d** A schematic presentation of CCB coordination by GLU4. Van der Waals contacts are indicated by black dashed curves, and hydrophilic interactions are indicated by cyan dashes.

In the structure of GLUT4, the amino terminal domain (NTD, containing TMs 1-6) and carboxyl terminal domain (CTD, containing TMs 7-12) enclose a large cavity that opens to the intracellular side, a state defined as inward-open (Fig. 2a, left). The inhibitor CCB is known to inhibit GLUTs with inward-open conformation^30,31^, which is consistent with our present structure. CCB is accommodated in the big cleft between the NTD and CTD (Fig. 2a, right upper panel). CCB comprises three ring structures, a macrolide ring, a nine-membered bicyclic ring, and a phenyl ring, all of which are well-resolved in the cryo-EM map (Fig. 2a, right lower panel).

The macrolide ring of CCB is coordinated by Asn176 and Trp404 through polar interactions, and Phe38, Trp404, and Trp428 through hydrophobic interaction (Fig. 2b, c). The bicyclic ring is nestled in a hydrophobic cavity constituted by Ile42, Ile180, Ile184, Ile303, Phe307, and Phe395. The coordination is buttressed by hydrogen bonds with Gln298, Gln299, and Trp404. The phenyl ring interacts with Ile180, Gln177, and Pro401 via hydrophobic contacts (Fig. 2b–d).

### Comparison of inward-open GLUT4 and GLUT1

**Fig. 3:**
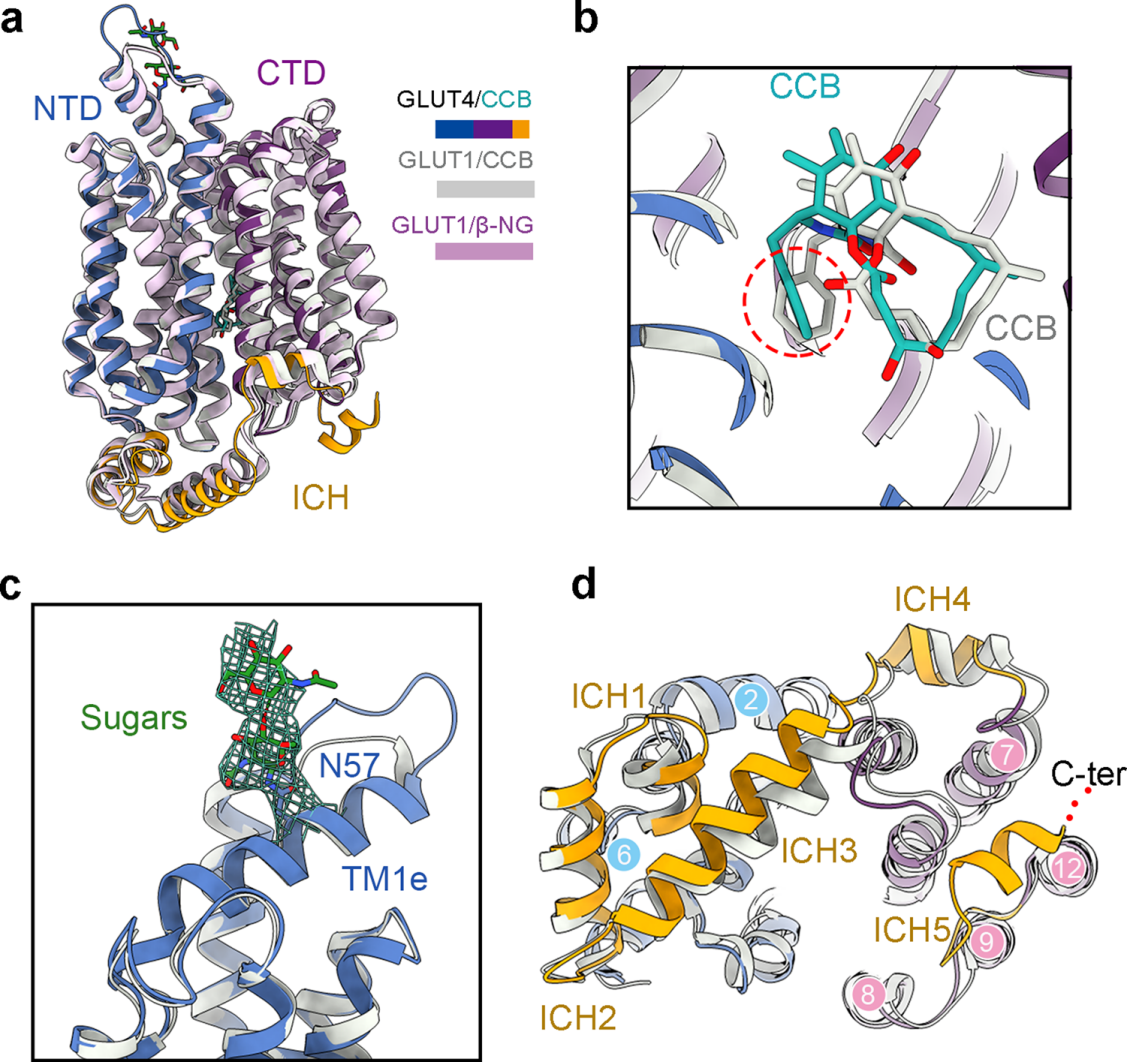
Structural comparison of inward-open GLUT4 and GLUT1. **a** Structural comparison of GLUT4 and GLUT1. Shown here are superimposed structures of GLUT4 (domain colored) with GLUT1 bound to CCB (silver, PDB code: 5EQI) or in the presence of β-NG (pink, PDB code: 4PYP). The TM region remains nearly identical. **b** Similar coordination of CCB by GLUT4 and GLUT1. Among all the CCB-coordinating residues, there are only two varied residues, Ile42 and Asn176 in GLUT4 are respectively substituted with Thr30 and His160 in GLUT1. **c** Cryo-EM analysis reveals the glycosylation site on GLUT4. The density for two sugar moieties (green) attach to Asn57 on the extracellular helix TM1e of GLUT4 is contoured at 4 σ. **d** Deviations of the ICH domain between GLUT4 and GLUT1. In contrast to the nearly identical structures of the TM region, there are deviations of ICH3 and ICH4 between GLUT4 and GLUT1. More importantly, the C-terminal ICH5 is resolved in GLUT4 only.

We next compared GLUT4 to GLUT1, whose structures have also been captured in the inward-open state in the presence of CCB (PDB code: 5EQI) or β-NG (PDB code: 4PYP)^12,31^. The cryo-EM structure of GLUT4 can be superimposed to the crystal structures of GLUT1-CCB and GLUT1-NG with RMSD values of 1.13 Å and 1.09 Å over 439 and 435 aligned Cα atoms, respectively, with identical transmembrane domains (Fig. 3a). Among all the CCB-coordinating residues, two loci are different between the two proteins; Ile42 and Asn176 in GLUT4 are respectively substituted with Thr30 and His160 in GLUT1. The phenyl ring of CCB rotates by about 60 degrees in the two structures (Fig. 3b).

GLUT1-4 all contain a conserved N-linked glycosylation site on an extracellular helix designated TM1e, which is a bent extension of TM1. To facilitate crystallization, the glycosylation site in GLUT1, GLUT3, and GLUT5 was eliminated by single point mutation or deglycosylation treatment^12,13,31,32^. In the cryo-EM map for GLUT4, a characteristic glycan density is observed contiguous with Asn57, exemplifying the unique power of cryo-EM in resolving posttranslational modifications (Fig. 3c).

Other than the observation of the extracellular glycosylation, ICH exhibits the most evident differences between GLUT4 and GLUT1. Our previous structural analysis of GLUT1 and GLUT3 defined five ICH helices, ICH1-ICH4 between the N and C domains and ICH5 after TM12^12,13^. ICH3 and ICH4 display minor positional shifts between GLUT4 and GLUT1 (Fig. 3d). The major distinction occurs to ICH5, which is invisible in the structure of GLUT1, but clearly resolved in the EM map of GLUT4 (Fig. 3d and Supplementary Fig. 6). We examined the functional role of ICH5 based on the structural comparison of outward-facing GLUT3 and inward-open GLUT4.

### ICH5 is important for the transport activity of GLUTs

**Fig. 4:**
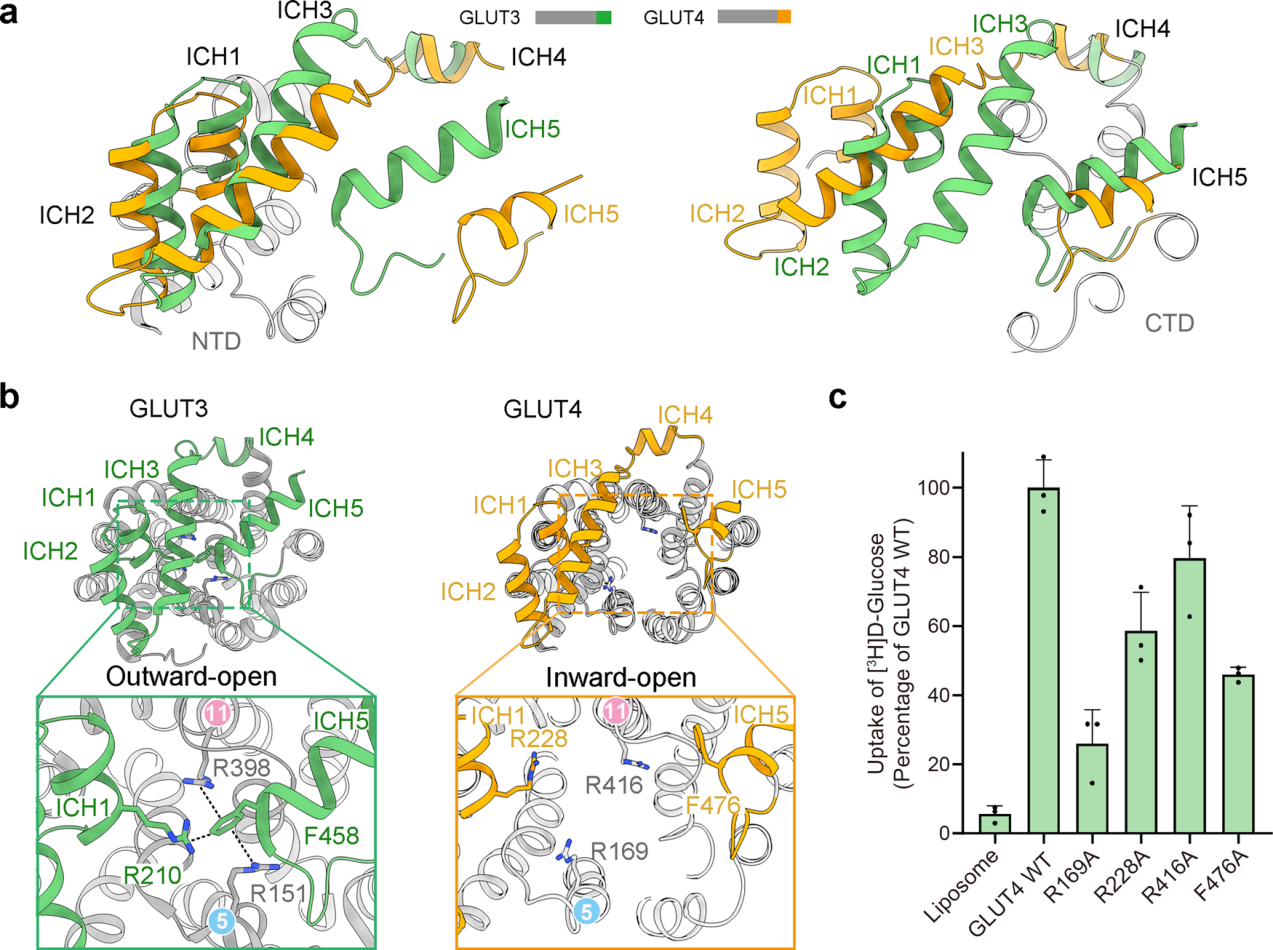
The ICH domain contributes to the intracellular gating during alternating access cycle of GLUTs. **a** Conformational changes of the ICH domain between the outward-occluded GLUT3 and inward-open GLUT4 structures. The cryo-EM structure of GLUT4 is superimposed to the crystal structure of GLUT3 (PDB code: 4ZW9) relative to the N (left) and C (right) domains, respectively. **b** A conserved Phe residue on ICH5 serves as a local organizing center in the outward-facing state. left, Phe458 on helix ICH5 forms cation-π interactions with multiple Arg residues in the N and C domains of outward-facing GLUT3. right, Rearrangement of the ICH domain in inward-open GLUT4. Phe476 in GLUT4 (equivalent of Phe458 in GLUT3) no longer interacts with any of the Arg residues in the inward-open state. **c** The interaction between Phe458 and N-domain Arg residues contributes to the transport activity of GLUTs. Shown here are normalized transport activities of GLUT4 variants in the liposome-based counterflow assay. Data are presented as mean with standard deviation, in three independent experiments. Source data are provided as a Source Data file.

The ICH domain undergoes major rearrangement between the outward-facing structure of GLUT3 and inward-open structure of GLUT4 (Fig. 4a). In the structure of GLUT3 (PDB code: 4ZW9), the ICH domain serves as a latch to secure the closure of the N and C domains on the intracellular side. Phe458 on ICH5 is surrounded by three Arg residues, Arg151 on TM5, Arg210 on ICH1, and Arg398 on TM11 (Fig. 4b, left). These residues are invariant between GLUT3 and GLUT4 (Supplementary Fig. 1). As ICH5 was invisible in previous structures, we did not get a chance to examine the rearrangement of this cation-π cluster. In the cryo-EM map for GLUT4, Phe476 (corresponding to Phe458 in GLUT3) is clearly resolved (Supplementary Fig. 6). The cation-π network is completely disassembled with ICH5 moving away (Fig. 4b, right).

To examine the functional relevance of this intracellular cluster, we substituted each of the Arg residues and Phe476 of GLUT4 with Ala and performed counterflow assay for these variants (Fig. 4c). GLUT4-F476A only retained half of the transport activity of WT, supporting an important role of this ICH5 residue in the alternating access transport process of GLUT4. Ala replacement of Arg169 (equivalent of GLUT3-Arg151), which also interacts with a Glu residue on TM10 and two backbone carbonyl oxygen groups on the loop between TM12 and ICH5, lost more than 70% of the activity. Mutation of Arg228 (equivalent of GLUT3-Arg210), which is mainly involved in bridging ICH1 and ICH3 other than binding to Phe476, reduced the activity by ~40%. Mutation of Arg416 has the least effect on the transport activity, with a reduction of approximately 20%. This result is consistent with the C domain-localization of Arg416, as there is barely any relative motion between the C domain and ICH5 during the alternating access cycle (Fig. 4a). The assay results are consistent with the structural indication that ICH5 is important for the transport activity of GLUTs.

## Discussion

GLUT4 plays a key role in reducing the glucose level in blood and insulin resistance. Insulin stimulates glucose uptake by acutely upregulating GLUT4 surface levels mainly through increased exocytosis of GSVs and decreased endocytosis^33,34^. A single GLUT4 molecule during its lifetime goes through multiple cycles of exocytosis and endocytosis^11^. Structural resolution of the WT full-length human GLUT4 lays an important framework for further investigation of GLUT4 regulation by insulin signaling.

**Fig. 5:**
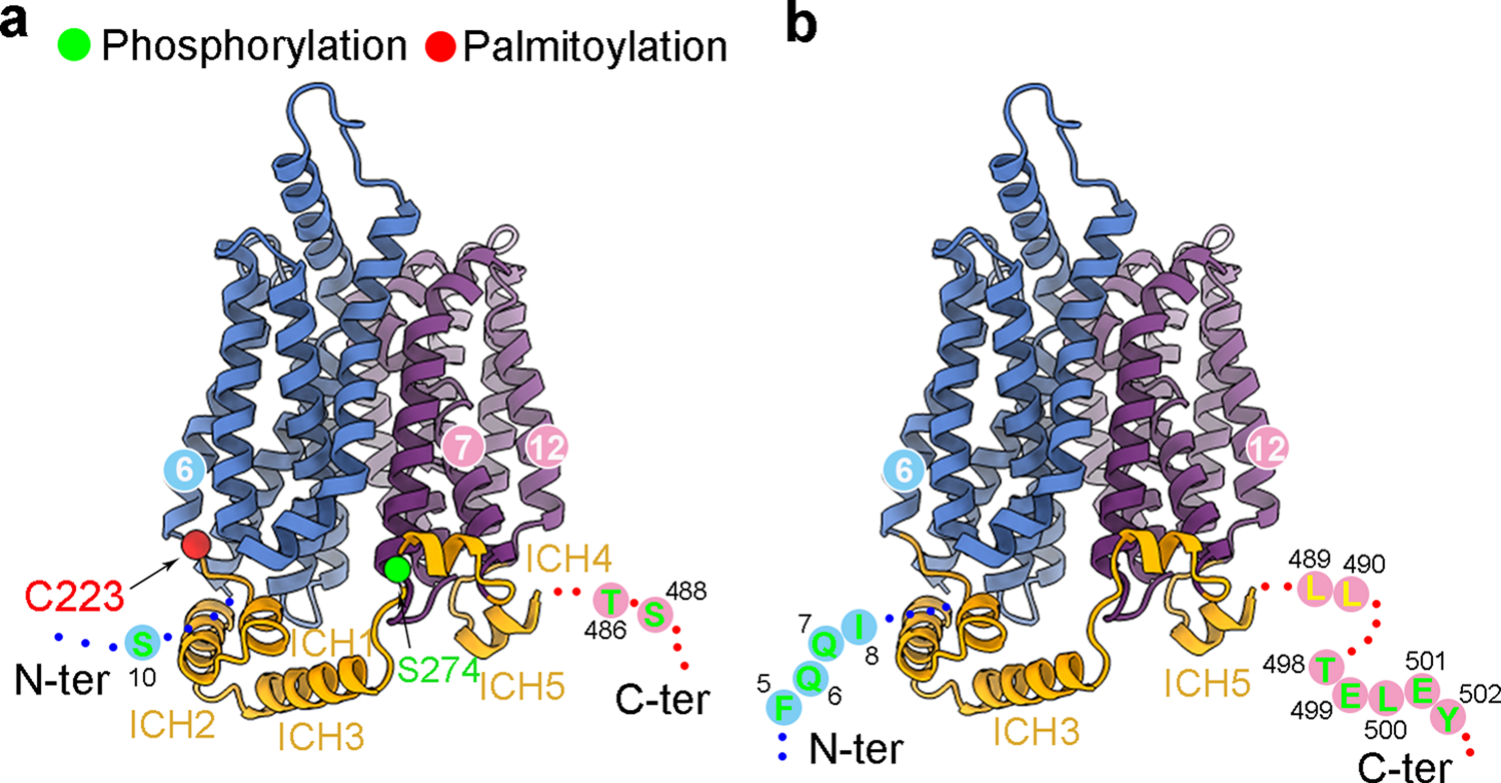
The intracellular functional motifs on GLUT4. **a** Posttranslational modification site on the intracellular segments of GLUT4. Residues that may be subject to phosphorylation and palmitoylation are shown as green and red spheres. Three potential phosphorylation sites, Ser10, Thr486, and Ser488, are invisible. **b** The intracellular motifs that are involved in the cellular trafficking of GLUT4 are localized on the flexible terminal loops that are not resolved in the cryo-EM structure. This arrangement may ensure cargo recognition independent of the functional states of GLUT4.

The cellular trafficking of GLUT4 has been shown to be regulated by posttranslational modifications. Cys223, which is at the end of the intracellular end of TM6, can be palmitoylated by DHHC7, an event that controls insulin-dependent translocation of GLUT4 to the plasma membrane^35^ (Fig. 5a). Future structural determination of palmitoylated GLUT4 may reveal the molecular basis for this regulation.

Four Ser residues, Ser10, Ser274, Thr486, and Ser488^36–38^, are subject to phosphorylation. Among these, only Ser274 is resolved to be positioned immediately preceding ICH4, while the other three are on the invisible N or C terminal loops (Fig. 5a and Supplementary Fig. 1). Note that Ser488 is right before the LL motif that mediates interaction with adaptor proteins^39^. Such arrangement provides a clue to the regulation of GLUT4 by Ser488 phosphorylation.

None of the GLUT4-unique sequences, _5_FQQI_8_, _489_LL_490_, and _498_TELEY_502_, is visible, likely owing to the intrinsic flexibility of these segments (Fig. 5b). These motifs have been reported to interact with proteins like Golgi-localized γ-ear-containing ARF-binding protein (GGA), retromer, and AP1 adaptor complexes^14–18,40–43^. Positioning of these motifs on the flexible termini ensures that their recognition by the adaptor proteins is independent of the transporter conformation.

In sum, our structural determination of intact human GLUT4 once again exemplifies the power of cryo-EM. It suggests that many other small size SLCs that have pseudo or no symmetry, can be directly analyzed by cryo-EM without the need for screening binders. Our study also marks an important step forward towards the mechanistic understanding of GLUT4 regulation by insulin signaling.

## Methods

### Protein expression and purification

Human GLUT4 (UniProt ID: P14762) cDNA or mutations were cloned into a pCAG vector with an N-terminal FLAG tag. 2 mg plasmid and 4 mg polyethylenimine (Polysciences) were preincubated in 50 ml fresh SMM 293-TII medium (Sino Biological) for 30 min before adding into one litre HEK293F cells at density of 2.0 × 10^6^ cells per ml. After 48 h incubation at 37 °C under 5% CO_2_, cells were harvested and resuspended in the buffer containing 25 mM HEPES pH 7.4, 150 mM NaCl, 5 μg/ml aprotinin, 1 μg/ml pepstatin, and 5 μg/ml leupeptin. Cell membrane was solubilized with 2% (w/v) *n*-dodecyl-β-D-maltoside (DDM, Anatrace) at 4 °C for 2 h. After high-speed centrifugation at 20,000 *g* for 30 min, the supernatant was loaded onto anti-FLAG M2 resin (Sigma). Then the resin was rinsed with wash buffer containing 25 mM HEPES pH 7.4, 150 mM NaCl, and 0.02% (w/v) DDM. Protein was eluted with wash buffer plus 0.4 mg/ml FLAG peptide. Elution was subjected to Superdex 200 Increase 10/300 GL column (GE Healthcare) in buffer containing 25 mM HEPES pH 7.4, 150 mM NaCl, and 0.1% (w/v) *n*-nonyl-β-D-glucopyranoside (β-NG, Anatrace). Peak fractions were pooled for further experiments. For purification in other detergents, the detergent was changed from wash buffer to 0.02% (w/v) glyco-diosgenin (GDN, Anatrace), 0.01% (w/v) lauryl maltose neopentyl glycol (LMNG, Anatrace) plus 0.01% (w/v) cholesteryl hemisuccinate tris salt (CHS, Anatrace), 0.02% (w/v) DDM plus 0.02% (w/v) CHS, respectively.

The membrane scaffold protein MSP1D1 with N-terminal 6 × His tag was expressed in *E. coli* BL21(DE3) cells. Collected cells were lysed by sonication in lysis buffer (25 mM Tris-HCl pH 8.0, 150 mM NaCl) supplemented with 5 μg/ml aprotinin, 1 μg/ml pepstatin, and 5 μg/ml leupeptin. After centrifugation at 20,000 *g* for 30 min at 4 °C, the supernatant was loaded onto the nickel-affinity resin. The resin was washed with lysis buffer plus 20 mM imidazole. Protein was eluted with lysis buffer plus 300 mM imidazole. The flow-through was concentrated and loaded onto a Superdex 200 Increase column equilibrated with lysis buffer. The MSP1D1-containing fractions were pooled for nanodisc reconstitution.

### Reconstitution of GLUT4 into lipid nanodiscs

1-palmitoyl-2-oleoyl-sn-glycero-3-phospho-(1’-rac)-choline (POPC, Avanti Polar Lipids) and 20% (w/w) cholesterol solubilized in chloroform was dried under nitrogen gas and resuspended with 25 mM HEPES, pH 7.4, 150 mM NaCl, 0.7% (w/v) DDM. GLUT4, MSP1D1 and lipid mixture were mixed at a molar ratio of 1:5:60 and incubated at 4 °C for 3 h. Detergents were removed by incubation with Biobeads SM2 (Bio-Rad) overnight at 4 °C. The protein lipid mixture was loaded onto a size-exclusion column equilibrated with 25 mM HEPES pH 7.4, 150 mM NaCl. The purified nanodiscs were collected for cryo-EM sample preparation.

### Preparation of liposomes and proteoliposomes

*E. coli* polar lipid extract (Avanti) was resuspended to 20 mg/ml in KPM buffer (50 mM potassium phosphate pH 6.5, 2 mM MgSO_4_) plus 50 mM D-glucose. After pre-incubation with 1% *n*-octyl-β-D-glucopyranoside (β-OG, Anatrace) for 30 min, liposomes were incubated with 200 μg/ml GLUT4, WT or mutants for 1 h at 4 °C. Detergents were removed by incubation overnight with 400 mg/ml Bio-Beads SM2. Proteoliposomes were collected by ultracentrifugation at 100,000 *g* for 1 h and resuspended to 100 mg/ml with KPM buffer for counterflow assay.

### Counterflow assay

Each counterflow assay was performed by adding 2 μL proteoliposomes into 98 μL KPM buffer containing 1 μCi (0.427 μM) D-[2-^3^H]-glucose (PerkinElmer). After incubation for 30 s, solution was aspirated through 0.22 μm GSTF filter (Millipore). Then the filter was rinsed with 2 mL ice-cold KPM buffer and incubated with 0.5 mL Optiphase HISAFE 3 (PerkinElmer) overnight for liquid scintillation counting.

For the measurement of *K*_m_ and V_max_, unlabeled glucose was added into KPM buffer at the indicated concentration, and the initial velocities were measured in 15 s. For the measurement of IC_50_ value, proteoliposomes were preincubated with the indicated concentrations of CCB (Sigma) for 30 min before adding into reaction buffer. All experiments were repeated for three times and data were examined by GraphPad Prism. Error bars represent SD.

### Cryo-EM sample preparation and data acquisition

For detergent sample, GLUT4 protein concentrated to approximately 8 mg/ml was incubated with 1 mM CCB for 30 min at 4 °C. Grids (Quantifoil Au R1.2/1.3, 300 mesh) were glow-discharged for 45 s using the medium power setting of the Plasma Cleaner PDC-32G (Harrick). For nanodisc sample, protein at 1.8 mg/ml was incubated with 1 mM CCB for 30 min at 4 °C. Grids coated with a thin layer of homemade graphene film were glow-discharged for 12 s using the low power setting.

4 μL aliquots of the sample were applied to grids and blotted for 3.0 s before being flash-frozen in liquid ethane cooled by liquid nitrogen with Vitrobot Mark IV (Thermo Fisher Scientific) at 8 °C and 100% humidity. The grids were transferred to a 300 kV Titan Krios equipped with Gatan K3 Summit detector and a GIF Quantum energy filter (slit width 20 eV). Micrographs were recorded in a defocus range from −2.0 to −1.5 μm. Each stack of 32 frames was exposed for 2.56 s, with an exposing time of 0.08 s per frame. The total dose was about 50 e^-^/Å^2^ for each stack. AutoEMation was used for the fully automated data collection^44^.

### Data processing

All 32 frames in each stack were aligned and summed using the whole-image motion correction program MotionCor2^45^ and binned to a pixel size of 1.083 Å. Dose-weighted micrographs were used for CTF estimation using Patch-CTF in cryoSPARC^28^. Micrographs with CTF fitting resolution worse than 4 Å were excluded during manual curation. Initial particles were picked from few micrographs using blob picker in cryoSPARC^28^ and 2D averages were generated. Final particle picking was done by template picker using templates from those 2D results. Particles were extracted using a box size of 192 pixels and cropped into 128 pixels to speed up early calculation steps. For simplicity, we will hereafter refer to the three datasets for GLUT4 in β-NG, LMNG/CHS and nanodisc as the β-NG, LMNG/CHS and nanodisc datasets, respectively.

The β-NG dataset was used for initial reference generation. 3578 k particles were extracted from 3131 micrographs with pixel size of 1.623 Å. After two rounds of 2D classification, ab-initio reconstruction, and hetero refinement for guided multi-reference 3D classification, a 6 Å map with clear transmembrane helices was generated. The remaining 430 k particles were used as seeds for seed-facilitated classification^26^. After that, 808 k particles were re-extracted with pixel size of 1.083 Å. Ab-initio reconstruction for non-reference 3D classification and hetero refinement for guided multi-reference 3D classification were carried out on these particles alternatively, yielding 303 k good particles. After Non-uniform refinement and local refinement, the β-NG dataset gave rise to a reconstruction at an average resolution of 4.11 Å (Supplementary Fig. 3).

For the LMNG/CHS dataset, 4154 k particles were extracted from 2552 micrographs with pixel size of 1.624 Å. After three rounds of hetero refinement for guided multi-reference 3D classification, 754 k good particles that just generated were used as seed for seed-facilitated classification. After that, 787 k particles were re-extracted with pixel size of 1.083 Å. Ab-initio reconstruction and hetero refinement were carried out alternatively for 3D classification, yielding 260 k particles. After Non-uniform refinement and local refinement, the final reconstruction reached 3.31 Å (Supplementary Fig. 4).

A similar procedure was carried out for nanodisc dataset, which finally, yielding a reconstruction at the resolution of 3.25 Å (Supplementary Fig. 5). The angular distributions of the particles used for the final reconstruction of all three datasets are reasonable (Supplementary Figs. 3c, 4c, and 5c).

### Model building and refinement

An initial structure model for GLUT4 was generated by AlphaFold2^46^. The structure was docked into the density map and manually adjusted and re-built by COOT^47^. The coordination of CCB was imported from GLUT1 model (PDB code: 5EQI) and restraint file for refinement was generated by PHENIX^48,49^.

The final models of GLUT4 for LMNG/CHS and nanodisc datasets were refined against the corresponding maps using PHENIX in real space with secondary structure and geometry restraints^48^. Overfitting of model was monitored by refining the model in one of the two independent maps from the gold-standard refinement approach, and testing the refined model against the other map^50^ (Supplementary Fig. 6b, c). The structures of GLUT4 were validated through examination of the Molprobity scores^51^ and statistics of the Ramachandran plots (Supplementary Table 2).

### Reporting summary

Further information on research design is available in the Nature Research Reporting Summary linked to this article.

## Supplementary information

Supplementary Information

Peer Review File

Reporting Summary

## Data Availability

The data that support this study are available from the corresponding authors upon reasonable request. The cryo-EM maps have been deposited in the Electron Microscopy Data Bank (EMDB) under accession number EMD-32760 (GLUT4 bound to CCB in nanodisc), EMDB-32761 (GLUT4 bound to CCB in LMNG/CHS) and the associated models have been deposited in the RSCB Protein Data Bank (PDB) under accession number 7WSM (GLUT4 bound to CCB in nanodisc), 7WSN (GLUT4 bound to CCB in LMNG/CHS). Previously solved structures have been deposited in PDB under the accession code 4PYP (GLUT1 structure in the presence of β-NG), 5EQI (GLUT1 structure in the presence of CCB), and 4ZW9 (GLUT3 structure in the presence of glucose). Source data are provided with this paper.

## Source data

Source Data
