# Supplementary material for: Cryo-EM structure of human glucose transporter GLUT4
Source: Nat Commun. 2022 May 13;13:2671. doi: 10.1038/s41467-022-30235-5 (PMC9106701; doi:10.1038/s41467-022-30235-5)
Supplement: Supplementary file 1 — Supplementary Information [file 41467_2022_30235_MOESM1_ESM.pdf]

Supplementary Information

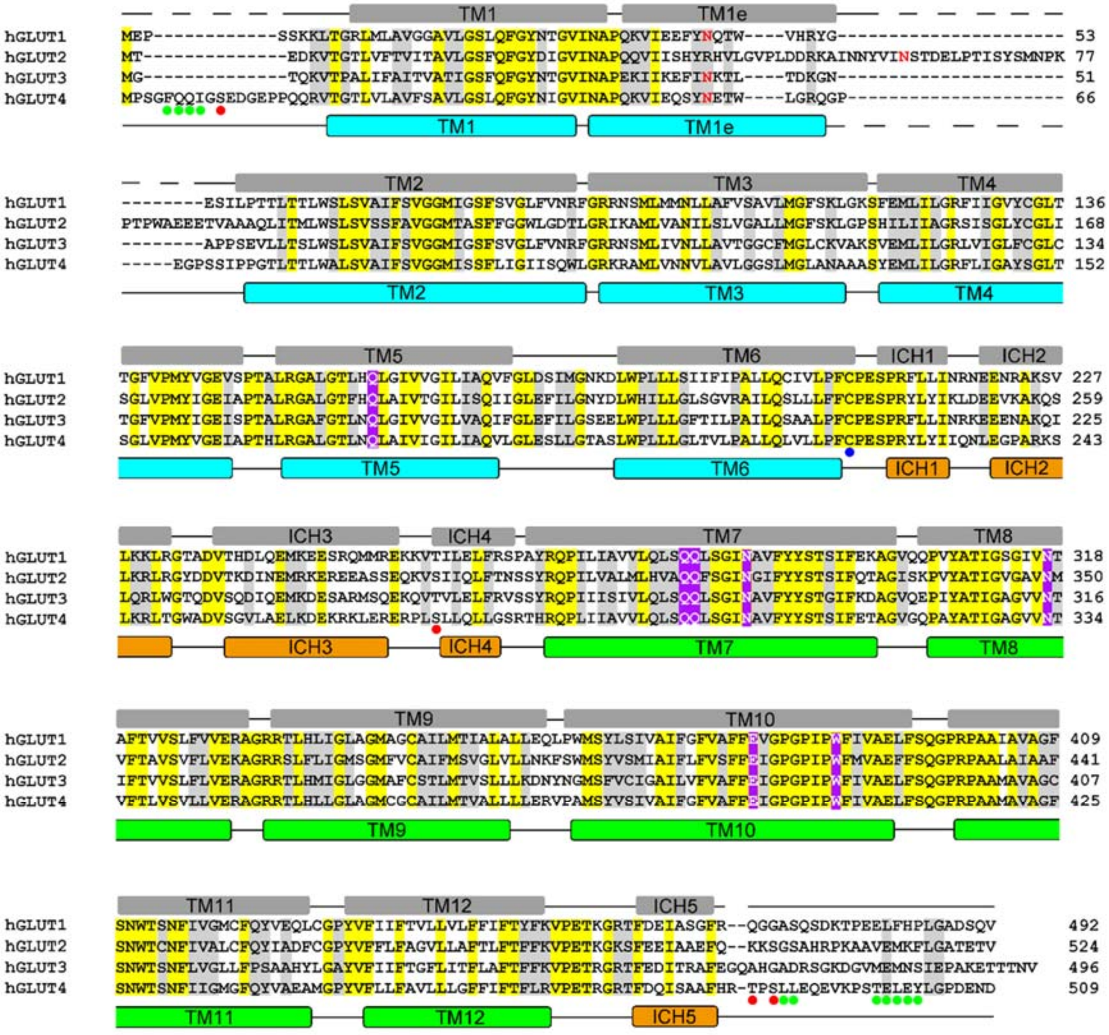

Supplementary Fig. 1 | Sequence alignment of human glucose transporters GLUT1-4. Secondary structural elements of GLUT1/GLUT4 are indicated above/below the sequence alignment. Invariant and highly conserved amino acids are shaded yellow and grey, respectively. The residues that are hydrogen-bonded to D-glucose in GLUT3 are shaded purple. Glycosylation residues are colored red. Residues related to phosphorylation in GLUT4 are indicated by red circles below. Cys223, which is subject to palmitoylation, is indicated by blue circles below. The unique sequences, including an FQQI motif on the amino (N) terminus and the LL and TELEY motifs on the carboxyl (C) terminus, are indicated by green circles below.

The Uniprot (https://www.uniprot.org) IDs for the aligned proteins are: hGLUT1 (P11166), hGLUT2 (P11168), hGLUT3 (P11169) and hGLUT4 (P14672). Sequences are aligned with ClustalW.

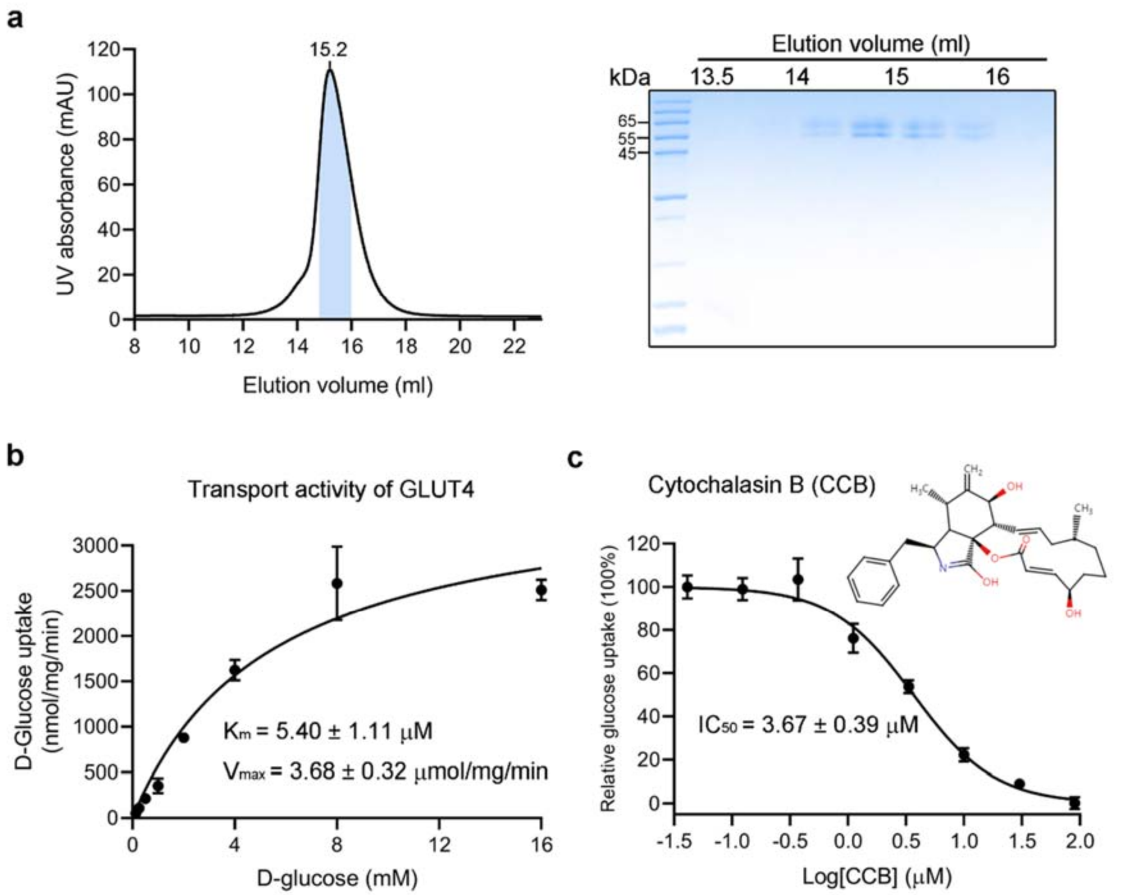

Supplementary Fig. 2 | Biochemical characterization of recombinantly expressed GLUT4. **a**, SEC purification of the human GLUT4 in the presence of 0.02% (w/v) DDM plus 0.002% (w/v) CHS (*left*) from one independent experiment. Peak fractions were further examined by Coomassie blue staining of SDS–PAGE (*right*). The fractions shaded blue in the left panel were pooled for cryo-EM analysis. **b**, Determination of the kinetic parameters of GLUT4 for the transport of D-glucose. The data were fitted using the Michaelis–Menten non-linear fitting method, yielding K_m at 5.40 ± 1.11 mM and V_{max} at 3.68 ± 0.32 μmol/mg /min. **c**, IC_{50} of the cytochalasin B for inhibition of GLUT4 transport. All data are presented as mean with SD of three independent experiments. Source data are provided as a Source Data file.

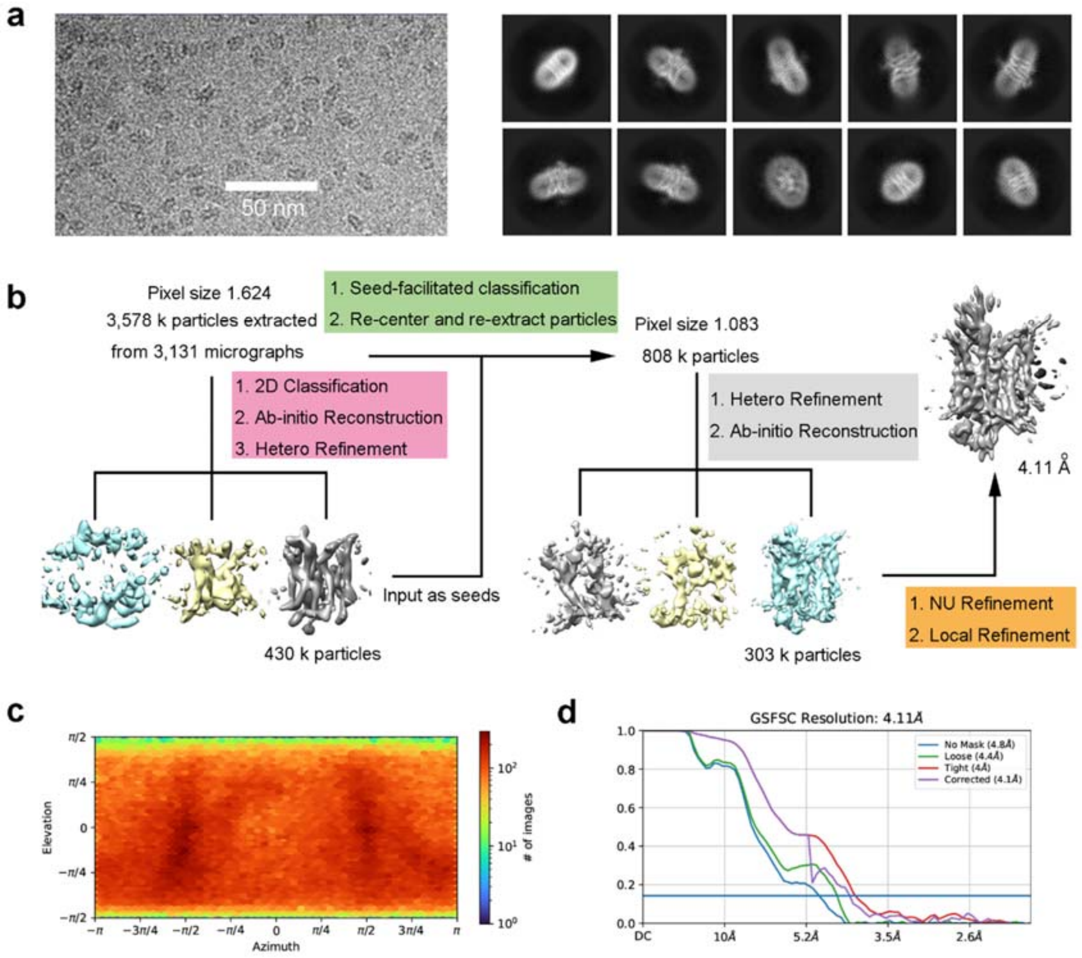

Supplementary Fig. 3 | Data processing of GLUT4 purified in β-NG. a, Representative micrograph and 2D class averages from one independent experiment. Transmembrane (TM) helices can be unambiguously visualized in the 2D averages. **b**, The flowchart for EM data processing. Details can be found in Materials and Methods. *Ab-initio* reconstruction is used for both initial reference generation and non-reference 3D classification and Hetero refinement is used for guided multi-reference 3D classification. Seed-facilitated 3D classification is used to improve the performance of 3D classifications for small membrane proteins. **c**, Angular distribution curve for the final refinement. **d**, Golden Standard Fourier Shell Correlation (GSFSC) curve for the final refinement.

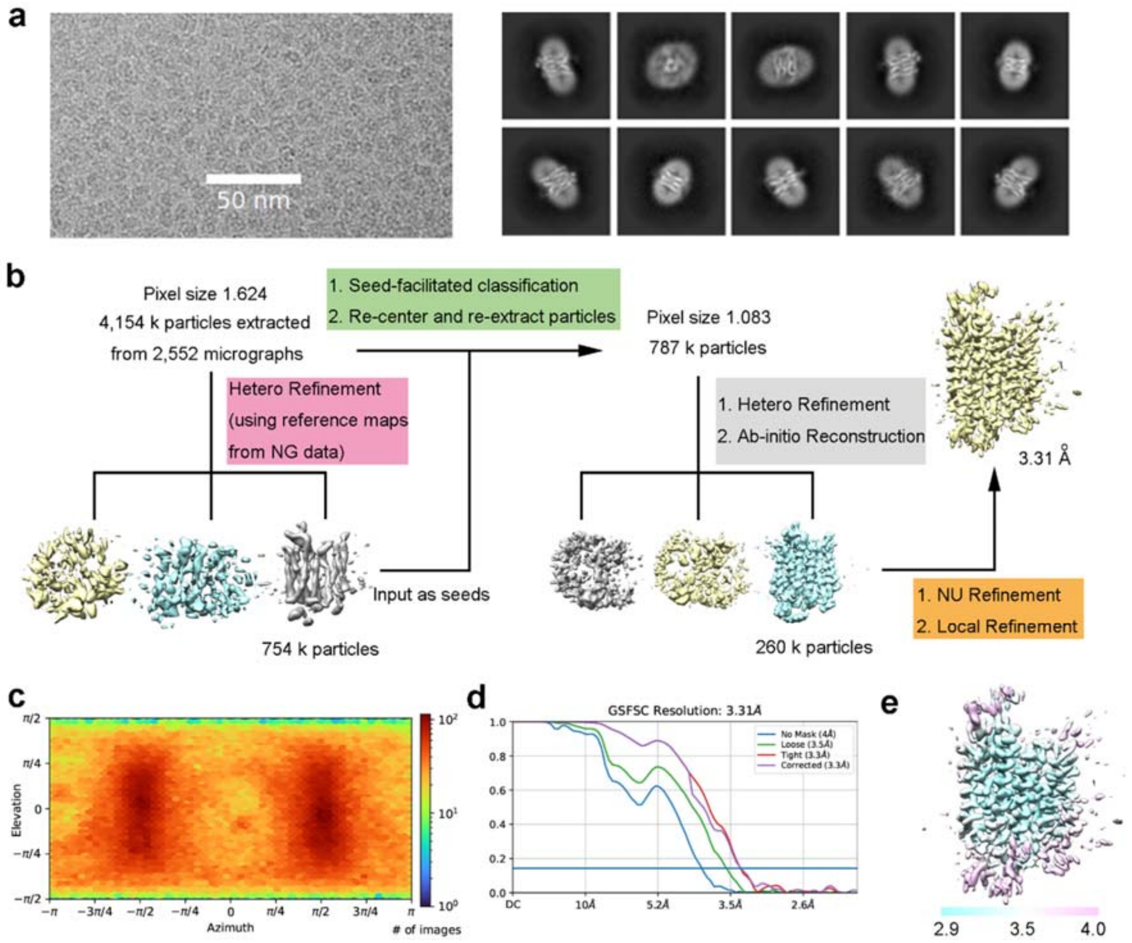

Supplementary Fig. 4 | Data processing of GLUT4 in LMNG/CHS. a, Representative micrograph and 2D class averages from one independent experiment. **b**, The flowchart for EM data processing. Details can be found in Materials and Methods. Reference maps used for initial classification on LMNG/CHS dataset come from NG dataset. **c**, Angular distribution curve for the final refinement. **d**, GSFSC curve for the final refinement. **e**, Local resolution of final map shows that protein core region reached 2.9 Å.

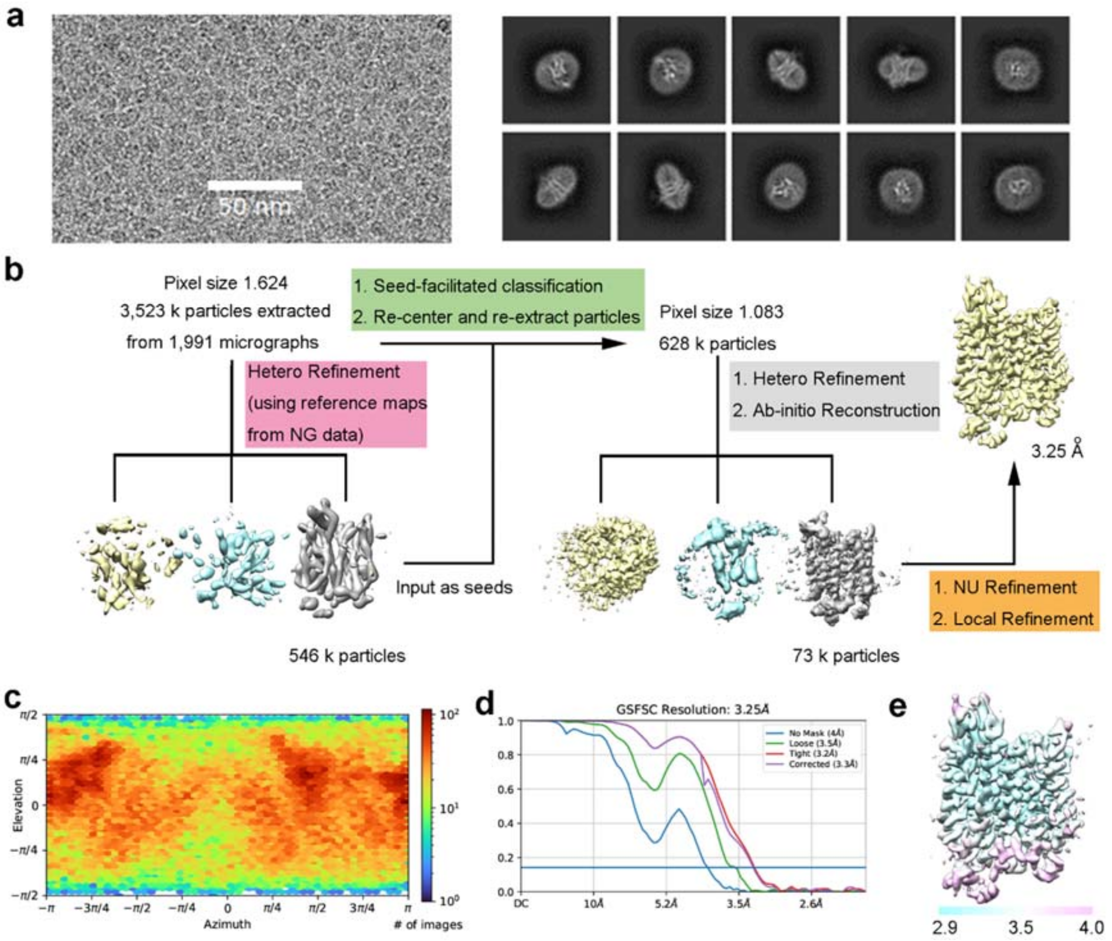

Supplementary Fig. 5 | Data processing of GLUT4 in nanodiscs. a, Representative micrograph and 2D class averages from one independent experiment. **b**, The flowchart for EM data processing. Details can be found in Materials and Methods. **c**, Angular distribution curve for the final refinement. **d**, GSFSC curve for the final refinement. **e**, Local resolution of final map shows that protein core region reached 2.9 Å.

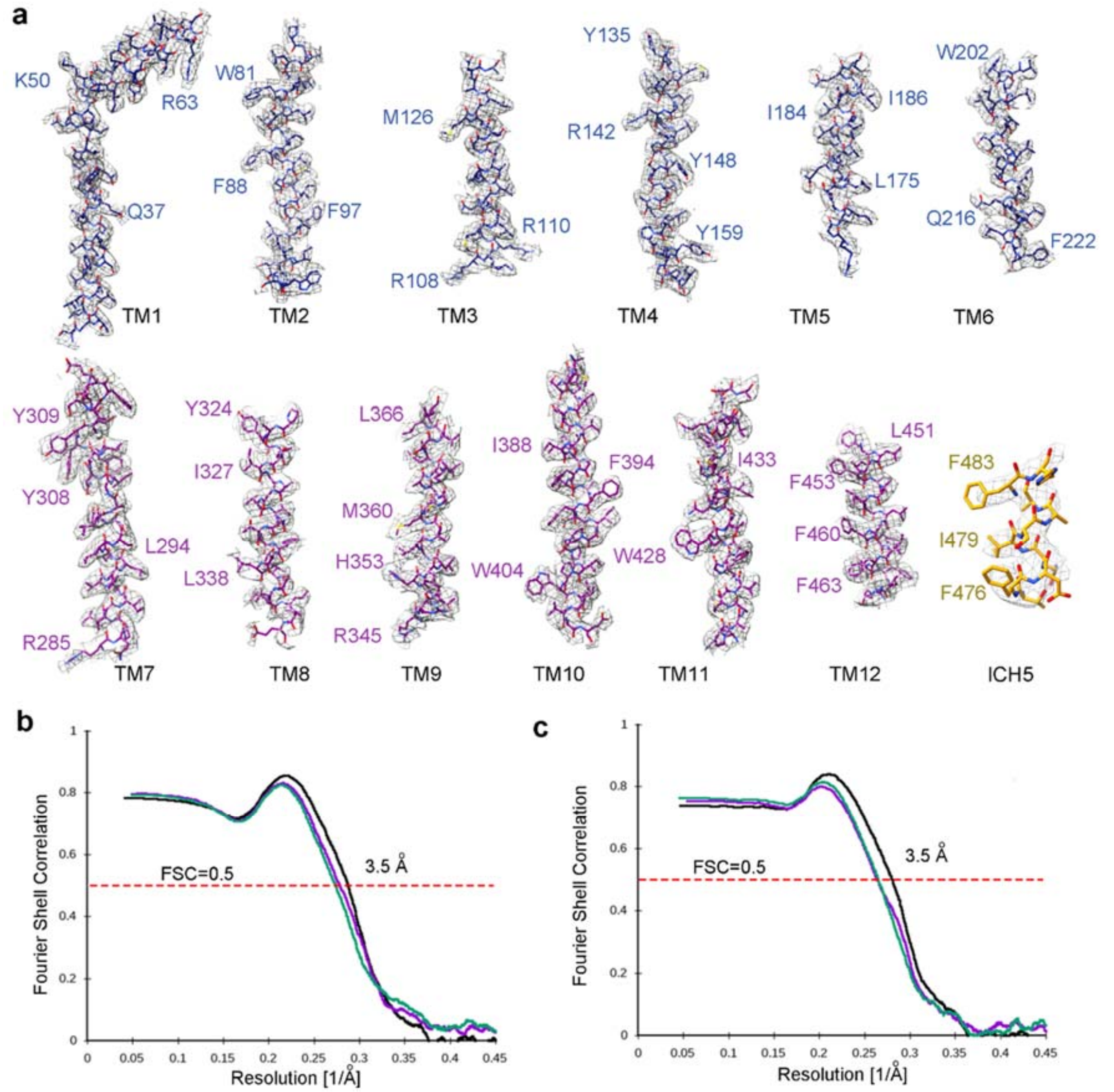

Supplementary Fig. 6 | Cryo-EM density of GLUT4 and model validation. a, EM maps for representative segments of GLUT4. Transmembrane helices in the NTD and CTD and ICH5 are colored blue, purple and yellow, respectively. The densities are contoured at 6 σ. **b**, **c**, Model validation for the LMNG/CHS dataset (left) and nanodisc dataset (right). FSC curves of the refined models versus refined maps are colored black. The models refined against refined maps versus the first half maps are colored purple. The models refined against the first half maps versus the second half maps are colored green. Differences between the purple curve and the green ones indicate that the refinement did not suffer from overfitting.

Supplementary Table 1 | Representative cryo-EM structures of SLC transporters.

SLC	Name	Year	Type	Species	Molecular Weight (kDa)	Total Structure Weight (kDa)	Resolution (Å)	EMDB code	PDB code
1A1	EAAT3	2021	Homotrimer	*Homo sapiens*	57	172	2.85	22011	6x2l
1A3	EAAT1	2021	Homotrimer	*Homo sapiens*	60	153	3.99	12524	7npw
1A4	ASCT1	2021	Homotrimer	*Homo sapiens*	56	167	4.2	13193	7p4i
1A5	ASCT2	2019	Homotrimer	*Homo sapiens*	57	170	3.37	12143	7bct
3A1	rBAT	2020	$b^{[0,+]}$AT1-rBAT complex	*Homo sapiens*	79	280	2.3	903	6li9
3A2	4F2hc	2019	LAT1-4F2hc complex	*Homo sapiens*	68	131	3.3	9721	6irs
4A4	NBCe1	2018	Homodimer	*Homo sapiens*	121	232	3.9	7441	6caa
5A1	SGLT1	2021	With nanobody	*Homo sapiens*	73	89	3.15	25196	7sla
5A2	SGLT2	2021	SGLT2-MAP17 complex	*Homo sapiens*	73	80	2.95	31558	7vsi
5A8	SMCT1	2021	With nanobody	*Homo sapiens*	67	82	3.5	25195	7sl9
6A4	SERT	2021	With Fab	*Homo sapiens*	70	89	3.3	23365	7lia
6A19	b^0AT1	2020	ACE2-b^0AT1 complex	*Homo sapiens*	71	345	2.9	30040	6m18
7A5	LAT1	2019	LAT1-4F2hc complex	*Homo sapiens*	55	131	3.3	9721	6irs
7A8	LAT2	2020	LAT2-4F2hc complex	*Homo sapiens*	58	134	2.9	30407	7cmi
7A9	$b^{[0,+]}$AT1	2020	$b^{[0,+]}$AT1-rBAT complex	*Homo sapiens*	53	280	2.3	903	6li9
7A11	xCT	2020	xCT-4F2hc complex	*Homo sapiens*	55	127	3.4	13267	7p9v
9A1	NHE1	2021	NHE1-CHP1 complex	*Homo sapiens*	91	102	3.3	30848	7dsw
9A9	NHE9	2020	Homodimer	*Equus caballus*	73	105	3.19	11067	6z3z
12A2	NKCC1	2019	Homodimer	*Danio rerio*	124	207	2.9	473	6npl
12A4	KCC1	2019	Homodimer	*Homo sapiens*	121	248	2.9	701	6kkr
12A5	KCC2	2021	Homodimer	*Homo sapiens*	126	254	3.2	30061	6m23
12A6	KCC3	2021	Homodimer	*Homo sapiens*	128	252	2.7	30058	6m22
12A7	KCC4	2020	Homodimer	*Homo sapiens*	119	248	2.9	30617	7d99
13A5	NaCT	2021	Homodimer	*Homo sapiens*	63	127	3.04	22457	7jsk

15A1	PepT1	2021	With soluble domain	*Homo sapiens*	79	79	3.5	13543	7pmx
15A2	PepT2	2021	With soluble domain	*Homo sapiens*	82	82	3.8	13544	7pmy
16A1	MCT1	2021	MCT1-Basigin2 complex	*Homo sapiens*	54	84	2.95	30389	7cko
16A7	MCT2	2020	Homodimer	*Homo sapiens*	52	108	3.8	30143	7bp3
17A6	VGLUT2	2020	With Fab	*Rattus norvegicus*	65	59	3.8	21040	6v4d
26A5	Prestin	2021	Homodimer	*Homo sapiens*	81	179	2.3	23329	7lgu
26A9		2020	Homodimer	*Homo sapiens*	87	174	2.6	30368	7ch1
28A3	CNT3	2020	Homotrimer	*Homo sapiens*	77	211	3.6	775	6ksw
30A8	ZnT8	2020	Homodimer	*Homo sapiens*	41	70	3.8	22285	6xpd
38A9		2020	SLC38A9-RagA-RagC-Ragulator complex	*Homo sapiens*	64	173	3.2	21686	6wj2
40A1	Ferroportin	2020	With Fab	*Homo sapiens*	63	118	2.5	21599	6wbv
46A1	PCFT	2021	With nanobody	*Gallus gallus*	50	65	3.2	12140	7bc6
59A1	MFSD2A	2021	With scFv	*Mus musculus*	60	59	3.5	24252	7n98
65A1	NPC1	2020	With soluble domain	*Homo sapiens*	142	151	3	21546	6w5s
65A2	NPC1L1	2021	With soluble domain	*Homo sapiens*	149	148	2.69	30666	7dfw
O6C1		2021	CatSpermasome	*Mus musculus*	79	915	2.9	31076	7eeb

Supplementary Table 2 | Cryo-EM data collection, refinement and validation statistics.

	Nanodisc<br>EMDB-32760<br>PDB:7WSN	LMNG/CHS<br>EMDB-32761<br>PDB:7WSM
**Data collection and processing**		
Magnification	81,000	81,000
Voltage (kV)	300	300
Electron exposure (e–/Å^2)	50	50
Defocus range (μm)	-2.0~-1.0	-2.0~-1.0
Pixel size (Å)	1.0825	1.0825
Symmetry imposed	C1	C1
Initial particle images (no.)	3,523 k	4,154 k
Final particle images (no.)	73 k	260 k
Map resolution (Å)	3.25	3.31
FSC threshold	0.143	0.143
Map resolution range (Å)	50~2.9	50~2.9
**Refinement**		
Initial model used (PDB code)	N/A	N/A
Model resolution (Å)	N/A	N/A
FSC threshold		
Model resolution range (Å)	N/A	N/A
Map sharpening *B* factor (Å^2)	111.7	157.5
Model composition		
Non-hydrogen atoms	3556	3556
Protein residues	464	463
Ligands	Cytochalasin B	Cytochalasin B
B factors (Å^2)		
Protein	72.47	85.08
Ligand	67.67	81.71
R.m.s. deviations		
Bond lengths (Å)	0.005	0.006
Bond angles (°)	0.698	0.749
Validation		
MolProbity score	1.86	1.95
Clashscore	8.21	11.25
Poor rotamers (%)	0.00	0.00
Ramachandran plot		
Favored (%)	94.57	94.13
Allowed (%)	5.43	5.87
Disallowed (%)	0.00	0.00
