# Supplementary material for: Cryo-EM structure of human glucose transporter GLUT4
Source: Nat Commun. 2022 May 13;13:2671. doi: 10.1038/s41467-022-30235-5 (PMC9106701; doi:10.1038/s41467-022-30235-5)
Supplement: Supplementary file 3 — Reporting Summary [file 41467_2022_30235_MOESM3_ESM.pdf]

# nature portfolio

Corresponding author(s): Chuangye Yan, Nieng Yan

Last updated by author(s): Apr 17, 2022



# Reporting Summary

Nature Portfolio wishes to improve the reproducibility of the work that we publish. This form provides structure for consistency and transparency in reporting. For further information on Nature Portfolio policies, see our Editorial Policies and the Editorial Policy Checklist.

## Statistics

For all statistical analyses, confirm that the following items are present in the figure legend, table legend, main text, or Methods section.

| n/a | Confirmed | |
|---|---|---|
| ☐ | ☒ | The exact sample size (*n*) for each experimental group/condition, given as a discrete number and unit of measurement |
| ☐ | ☒ | A statement on whether measurements were taken from distinct samples or whether the same sample was measured repeatedly |
| ☒ | ☐ | The statistical test(s) used AND whether they are one- or two-sided<br>*Only common tests should be described solely by name; describe more complex techniques in the Methods section.* |
| ☒ | ☐ | A description of all covariates tested |
| ☒ | ☐ | A description of any assumptions or corrections, such as tests of normality and adjustment for multiple comparisons |
| ☐ | ☒ | A full description of the statistical parameters including central tendency (e.g. means) or other basic estimates (e.g. regression coefficient) AND variation (e.g. standard deviation) or associated estimates of uncertainty (e.g. confidence intervals) |
| ☒ | ☐ | For null hypothesis testing, the test statistic (e.g. *F*, *t*, *r*) with confidence intervals, effect sizes, degrees of freedom and *P* value noted<br>*Give P values as exact values whenever suitable.* |
| ☒ | ☐ | For Bayesian analysis, information on the choice of priors and Markov chain Monte Carlo settings |
| ☒ | ☐ | For hierarchical and complex designs, identification of the appropriate level for tests and full reporting of outcomes |
| ☒ | ☐ | Estimates of effect sizes (e.g. Cohen's *d*, Pearson's *r*), indicating how they were calculated |

*Our web collection on statistics for biologists contains articles on many of the points above.*

## Software and code

Policy information about availability of computer code

| | |
|---|---|
| Data collection | AutoEMation v2 |
| Data analysis | GraphPad Prism v8.0, MotionCor2 v1, cryoSPARC v3.2, COOT v0.9.3, PHENIX v1.18, UCSF ChimeraX v1.2, AlphaFold v2 |

For manuscripts utilizing custom algorithms or software that are central to the research but not yet described in published literature, software must be made available to editors and reviewers. We strongly encourage code deposition in a community repository (e.g. GitHub). See the Nature Portfolio guidelines for submitting code & software for further information.

## Data

Policy information about availability of data

All manuscripts must include a data availability statement. This statement should provide the following information, where applicable:

- Accession codes, unique identifiers, or web links for publicly available datasets
- A description of any restrictions on data availability
- For clinical datasets or third party data, please ensure that the statement adheres to our policy

The cryo-EM maps for the structures of GLUT4 bound to CCB in LMNG/CHS and nanodiscs have been deposited in the Electron Microscopy Data Bank (EMDB) [https://www.ebi.ac.uk/emdb] (EMD-32760 and EMD-32761) and the associated models have been deposited in the RCSB Protein Data Bank (PDB) [https://www.rcsb.org] (7WSN and 7WSM). Previously solved structures have been deposited in PDB under the accession code 4PYP [http://doi.org/10.2210/pdb4PYP/pdb] (GLUT1 with β-NG structure), 5EQI [http://doi.org/10.2210/pdb5EQI/pdb] (GLUT1 with CCB structure), and 4ZW9 [http://doi.org/10.2210/pdb4ZW9/pdb] (GLUT3 with glucose structure), respectively.

# Field-specific reporting

Please select the one below that is the best fit for your research. If you are not sure, read the appropriate sections before making your selection.

- [x] Life sciences
- [ ] Behavioural & social sciences
- [ ] Ecological, evolutionary & environmental sciences

For a reference copy of the document with all sections, see nature.com/documents/nr-reporting-summary-flat.pdf

# Life sciences study design

All studies must disclose on these points even when the disclosure is negative.

| | |
|---|---|
| Sample size | No statistical methods was used to predetermine sample size. Three independent biological experiment were performed in liposome-based transport assay with similar results obtained. The sample particles of GLUT4 in detergents and nanodiscs were automatically picked from the collected cryo-EM micrographs by software packages. |
| Data exclusions | No data were excluded from the analyses. |
| Replication | Liposome-based transport assay were repeated for three independent experiments and successfully reproduced. |
| Randomization | As single particle analysis is automatically processed by software packages, randomization is not relevant to this study. |
| Blinding | Blinding was not applicable for this research. The initial cryo-EM micrographs were manually picked to exclude bad pictures with thick ice or low contrast. Investigators were not blinded to group allocation during data collection. |

# Reporting for specific materials, systems and methods

We require information from authors about some types of materials, experimental systems and methods used in many studies. Here, indicate whether each material, system or method listed is relevant to your study. If you are not sure if a list item applies to your research, read the appropriate section before selecting a response.

## Materials & experimental systems

| n/a | Involved in the study | |
|---|---|---|
| x | | Antibodies |
| | x | Eukaryotic cell lines |
| x | | Palaeontology and archaeology |
| x | | Animals and other organisms |
| x | | Human research participants |
| x | | Clinical data |
| x | | Dual use research of concern |

## Methods

| n/a | Involved in the study | |
|---|---|---|
| x | | ChIP-seq |
| x | | Flow cytometry |
| x | | MRI-based neuroimaging |

## Eukaryotic cell lines

Policy information about cell lines

| | |
|---|---|
| Cell line source(s) | HEK293F cell line was originally obtained from Invitrogen. |
| Authentication | None of the cell line used was authenticated. |
| Mycoplasma contamination | The cell line was not tested for mycoplasma contamination. |
| Commonly misidentified lines (See ICLAC register) | No commonly misidentified cell lines were used. |
